# The Trans-Kingdom Spectrum of Mpox-like Lesion Pustules of Suspect Patients in the Mpox Clade Ib Outbreak in Eastern Democratic Republic of the Congo

**DOI:** 10.3390/microorganisms13092025

**Published:** 2025-08-29

**Authors:** Leandre Murhula Masirika, Benjamin Hewins, Ali Toloue Ostadgavahi, Mansi Dutt, Léandre Mutimbwa Mambo, Jean Claude Udahemuka, Pacifique Ndishimye, Justin Bengehya Mbiribindi, Freddy Belesi Siangoli, Patricia Kelvin, Morgan G. I. Langille, David J. Kelvin, Luis Flores, Gustavo Sganzerla Martinez, Anuj Kumar

**Affiliations:** 1 Centre de Recherche en Sciences Naturelles de Lwiro, DS Bukavu, Sud-Kivu, Democratic Republic of the Congo; leandremurhula@gmail.com (L.M.M.); vet@lwiroprimates.org (L.F.); 2 SaBio Instituto de Investigación en Recursos Cinegéticos IREC, Universidade de Castilla-LaMancha & CSIC, Ronda de Toledo 12, 13005 Cuidad Real, Spain; 3 Congo Outbreaks, Research and Development, DS Bukavu, South-Kivu, Democratic Republic of Congo; 4 Department of Microbiology and Immunology, Canadian Center for Vaccinology (CCfV), Faculty of Medicine, Dalhousie University, Halifax, NS B3H 4R2, Canada; benjamin.hewins@dal.ca (B.H.); ali.toloue@dal.ca (A.T.O.); mansidutt@dal.ca (M.D.); patricia@bioforgecanada.ca (P.K.); gustavo.sganzerla@dal.ca (G.S.M.); kumaranuj@dal.ca (A.K.); 5 Laboratory of Immunity, Shantou University Medical College, Jinping, Shantou 512025, China; 6 BioForge Canada Limited, Halifax, NS B3N 3B9, Canada; 7 Zone de Santé de Kamituga, Kamituga, Sud-Kivu, Democratic Republic of the Congo; leandremambo@gmail.com; 8 Department of Veterinary Medicine, University of Rwanda, Nyagatare P.O. Box 57, Rwanda; jean.udahemuka@gmail.com; 9 Stansile Research Organization, Kigali P.O. Box 5377, Rwanda; ndipac@gmail.com; 10 Research and Innovation Centre, African Institute for Mathematical Sciences (AIMS), Kigali P.O. Box 6428, Rwanda; 11 Division Provinciale de la Santé, DS Bukavu, Sud-Kivu, Democratic Republic of the Congo; justinbengehya@gmail.com (J.B.M.); freddysiangoli15@gmail.com (F.B.S.); 12 Department of Pharmacology, Dalhousie University, Halifax, NS B3H 4R2, Canada; morgan.g.i.langille@dal.ca; 13 One Health Conservation Initiative, Katana, Kabare, Sud-Kivu, Democratic Republic of the Congo; 14 Centre de Rehabilitation des Primates de Lwiro, Lwiro, DS Bukavu, Sud-Kivu, Democratic Republic of the Congo

**Keywords:** mpox, clade Ib, metagenomics, co-infection

## Abstract

During infectious disease outbreaks, acquiring genetic data across various kingdoms offers essential information to tailor precise treatment methodologies and bolster clinical, epidemiological, and public health awareness. Metagenomics sequencing has paved the way for personalized treatment approaches and streamlined the monitoring process for both co-infections and opportunistic infections. In this study, we conducted long-read metagenomic DNA sequencing on mpox-like lesion pustules from six suspected patients who were positive and confirmed to be infected with MPXV during the MPXV subclade Ib outbreak in the Eastern Democratic Republic of the Congo. The sequenced data were taxonomically classified as bacterial, fungal, and viral in composition. Our results show a wide spectrum of microorganisms present in the lesions. Bacteria such as *Corynebacterium amycolatum*, *Gardnerella vaginalis*, *Enterococcus faecium*, *Enterobacter clocae*, *Staphylococcus epidermidis*, and *Stenotrophomonas maltophilia* were found in the lesions. The viral classification of the reads pointed out the absolute predominance of the monkeypox virus. Taken together, the outcomes of this investigation underscore the potential involvement of microorganisms in mpox lesions and the possible role that co-infections played in exacerbating disease severity and transmission during the MPXV subclade Ib outbreak.

## 1. Introduction

Mpox virus (MPXV), formerly known as monkeypox virus, is the causative agent of mpox and is endemic in regions of Central and East Africa, including the Democratic Republic of the Congo (DRC). In 1958, this orthopox virus was first time isolated in Denmark from an imported macaque (*Macaca fascicularis*), while the first confirmed human case was reported in a 10-year-old boy with a smallpox-like disease in the Equateur Province of the Democratic Republic of the Congo (DRC) in 1970 [1,2]. Since then, sporadic outbreaks of MPXV have increased in frequency [3]. Taxonomically, MPXVs are divided into two clades: clade I (formerly Central African or Congo Basin clade) and clade II (formerly West African clade). Despite the lack of standardized epidemiological reports that would allow clinical comparison of the human diseases caused by viruses of the two clades [4], emerging viruses from both clades have been able to cause sustained human-to-human transmission events.

MPXV is a zoonotic virus with a double-stranded DNA and belongs to the Orthopoxvirus genus of the Poxviridae family with a close association to other poxviruses, namely variola (VARV) and vaccinia viruses (VACV), which can also infect humans [5,6]. The MPXV genome is ~197 kb in length with covalently closed hairpin ends [7]. The genome is packed with the orthologous poxvirus genes (OPGs). These OPGs are distributed over the central conserved regions, also known as the core region and flanking terminal regions. Each end has a repeated DNA sequence about 6.4 kb long, terminal inverted repetitions (ITRs), which are flipped in opposite directions [5]. It has been reported that the MPXV genome encodes roughly 193 open reading frames (ORFs) with ≥60 amino acid (aa) residue proteins. “Housekeeping” gene encoding proteins of MPXV play a crucial role in replication and transcription. The OPGs are distributed over the central conserved region and significantly contribute to virion assembly. The OPGs located on the terminal regions of the MPXV genome encode a set of proteins that play an important role in the host range and pathogenesis [5]. Similar to other poxviruses, the MPXV genome also harbors several ITRs and nucleotide homopolymers [8,9]. Previously, short tandem repeats (STRs) have also been reported in the MPXV genome [9]. These STRs (dinucleotide, trinucleotide, or more complex palindromic repeats) have been reported to be distributed where more variation is reported [5].

In September 2023, sustained human-to-human transmission of the newly identified MPXV subclade Ib virus in Kamituga, a mining city in the province of South Kivu, DRC, led the World Health Organization (WHO) to declare a Public Health Emergency of International Concern on 14 August 2024 [10,11,12] as travel-related cases were observed in Europe, Asia, North America, South America, and Oceania, marking the first time MPXV clade I viruses were recorded outside the African continent. Since the reporting of the first confirmed cases, the virus has started to spread to the neighboring provinces and countries [10,11,13,14]. Clade Ib is now spreading globally, and a total of 33 countries have reported confirmed clade Ib cases from Africa (Angola, Burundi, Congo, the DRC, Ethiopia, Kenya, Malawi, Mozambique, Rwanda, South Africa, South Sudan, Uganda, the United Republic of Tanzania, Zambia, and Zimbabwe), Asia (China, India, Oman, Pakistan, Qatar, Thailand, and the United Arab Emirates), Europe (Belgium, France, Germany, Italy, Sweden, Switzerland, and the United Kingdom), North America (Canada and the United States of America), South America (Brazil), and Oceania (Australia), as of 31 July 2025 [15]. Of 33 countries, a total of 12 countries, namely the DRC, Uganda, Burundi, Kenya, Zambia, Rwanda, the United Republic of Tanzania, Congo, Malawi, Ethiopia, South Sudan, and Mozambique, have reported the sustained community transmission of clade Ib MPXV, while others reported travel-associated cases. In the past six months, the DRC, Uganda, Burundi, Kenya, Zambia, Rwanda, the United Republic of Tanzania, Congo, Malawi, Ethiopia, South Sudan, and Mozambique have reported a total of 1093, 791, 225, 118, 89, 02, 43, 12, 29, 08, 01, and 17 confirmed cases, respectively. Since January 2024, compared to other countries, a higher number of total confirmed cases of clade Ib MPXV—27,811, 7648, and 4231—have been reported from the DRC, Uganda, and Burundi, respectively [15].

The outbreak exhibited transmission dynamics not previously observed during other clade I mpox outbreaks, such as heterosexual transmission among professional sex workers (PSWs) and sustained (nonsexual) community-level transmission in adults, adolescents, and children [13]. Symptoms observed during this outbreak ranged from mild to severe and were characterized as a febrile illness with a progressive rash (macules, papules, vesicles, pustules, and scabbing) starting in the hands/feet, or genitals, and spreading over the body.

A detailed picture of the microbial makeup of mpox lesions during the subclade Ib outbreak is necessary to better understand why, specifically, this novel clade I virus is so efficiently transmitted between humans, a characteristic rarely documented in clade I mpox [16,17]. In much of Africa, mpox vaccines (Jynneos^®^, Bavarian Nordic, Copenhagen, Denmark, Imvamune^®^, Bavarian Nordic, Copenhagen, Denmark) are not readily available. Many available doses were preferentially procured and distributed among high-income countries during the 2022 global mpox clade IIb outbreak, placing many endemic regions in Africa at risk of ongoing and worsening outbreaks. Furthermore, routine screening for sexually transmitted illnesses (STI) in low- and middle-income countries (LMICs) in Africa, such as the DRC, is virtually nonexistent [18]. As the transmission of subclade Ib appears to be occurring via heterosexual contact (and community spread), the lack of STI screening exacerbates transmission. Compounding the previous issues in these settings is the lack of infrastructure, reagents and supplies, and trained personnel to perform testing/screening.

The role of co-infection and super-infection during mpox infection is well-documented. For example, a recent systematic review determined that of 6345 confirmed mpox cases, 40.32% of individuals (*N* = 2558) also had human immunodeficiency virus (HIV) co-infection [19]. The true number of HIV–mpox co-infected individuals in LMICs in Africa likely exceeds 40.32%, given that the majority of study locations reported in the review occurred in high-income countries, where contraception, STI screening, and access to healthcare is much greater. In addition to the reduced immune system function caused by HIV, and thus the ability to stave off additional diseases, patients with mpox are often diagnosed with other infections. These can include STIs (i.e., syphilis, chlamydia, and gonorrhea), herpes simplex virus (HSV), varicella zoster virus (VZV), cytomegalovirus, and species of *Mycoplasma* [20,21,22,23,24,25,26]. Given the morphological overlap of mpox, HSV, and VSV, broad STI screening and PCR should be incorporated as standard of care during suspected cases of mpox, where resources permit. Although genital mpox lesions in sexually active individuals are explained by sexual transmission, transmission during the subclade Ib outbreak is complicated by cases of community spread, where contact with infected materials (fomites) has been shown to spread the disease.

The metagenomics approach is one of the established methods for understanding microbial communities and studying their impact on human health. A metagenomics study has been reported for a common chronic inflammatory disease, namely acne vulgaris, where eligible participants were recruited, non-inflammatory (comedones) and inflammatory lesions (papules and pustules) were collected from face samples, and metagenomics was performed to study the skin microbiota [27].

Little is known regarding the viral, bacterial, and fungal communities in mpox-associated lesions. A greater understanding of these lesions may provide additional insights into the transmissibility, genetic diversity, and host specificity of novel clade Ib mpox. To address these gaps, we hypothesize that there is a wide spectrum of pathogens present in lesions caused by mpox, likely functioning synergistically to exacerbate the disease state and transmission. To test our hypothesis, we investigated the trans-kingdom spectrum of sequenced lesion samples to ascertain their viral, bacterial, and fungal composition.

## 2. Materials and Methods

### 2.1. Study Design and Sample Collection

A total of six samples were collected from lesion swabs obtained from suspected cases of mpox infection from five patients admitted to the Kamituga General Hospital in the city of Kamituga, South Kivu, Democratic Republic of the Congo. The DNA extraction was performed using the Qiagen DNeasy Blood & Tissue Kit (Qiagen, Hilden, Germany) according to the manufacturer’s instructions. The quality and quantity of DNA were determined using a Qubit 4 Fluorometer with the Qubit 1X dsDNA HS Assay Kit (Thermo Fisher Scientific, Waltham, MA, USA). The test was performed using a 1 µL sample volume according to the manufacturer’s protocol. For the successful preparation of libraries, the use of this high-sensitivity assay was crucial for accurate DNA quantification, even at low concentrations.

### 2.2. Metagenomics Sequencing Methodology

The sequencing libraries were prepared with two distinct kits—for R10 flow cells, the Rapid Sequencing DNA—PCR Barcoding Kit 24 V14 (SQK-RPB114.24), and for R9 flow cells, the Rapid PCR Barcoding Kit 24 (Version 14, SQK-RPB004)—both products of Oxford Nanopore Technologies (ONT), Oxford, UK. The procedure involved several steps including initial DNA tagmentation, barcode addition using rapid barcode primers, PCR amplification, and library cleanup with AMPure XP magnetic beads (Beckman Coulter, Brea, CA, USA), followed by a final quantification step with the Qubit 4 fluorometer. The libraries were then loaded on the R9 or R10 flow cells using proper flow cell priming and library loading procedures per ONT protocols. Sequencing runs were extended to 24 h with real-time base calling enabled through MinKNOW software (version 23.11.5, Oxford Nanopore Technologies) for live monitoring and data quality assessment. Nanopore raw reads were base-called using the MinKNOW software (ONT, version 23.11.5) with a minimum Q score of 8.0.

### 2.3. Taxonomic Annotation

Nanopore reads had their taxonomic composition annotated using Kraken2 (version 2.1.3) [28] using the PlusPF database, composed of RefSeq archaea, bacterial, viral, protozoa, and fungi (1 December 2024). The Kraken2 outputs were converted to a report file, which was carried over, read, and parsed in Python (version 3.12) to plot. In addition, the Kraken2 reports were visualized using the R package Pavian (version 1.2.1 running on R version 4.1.2).

## 3. Results

### 3.1. Metagenomics Sequencing

**Figure 1:**
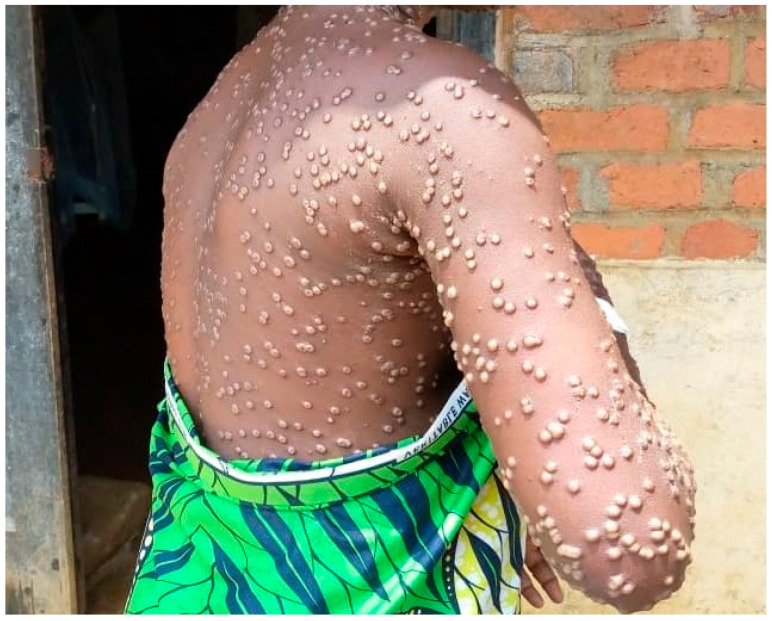
Representative lesions of the mpox outbreak in Kamituga.

Lesion swabs from six suspected mpox cases were obtained and sequenced using Oxford Nanopore Technologies. As shown in Table 1, these six samples were collected between 2 November 2023 and 14 January 2024, and their consensus genomes were subsequently deposited to the EpiPox resource of GISAID (Global Initiative on Sharing All Influenza Data) with the accession numbers EPI_ISL_19004044, EPI_ISL_19004045, EPI_ISL_19004046, EPI_ISL_19079342, EPI_ISL_19079343, and EPI_ISL_19079344. In Figure 1, we show a study participant, representative of the mpox Kamituga clade I subgroup VI, exhibiting multifocal, erythematous maculopapular mpox-like lesions distributed extensively across the left upper extremity, thoracic region, dorsal aspect, cervical region, and facial integument. Next, we show the results obtained from the long-read DNA sequencing of lesion swabs from six suspected mpox cases (Table 2). The number of raw reads varied from 964K to 3M. When considering reads that passed the minimum Q score of 8, the base-calling process generated from 3.18 to 10.45 giga bases. In addition, the raw reads were classified using Kraken2 to determine their metagenomic composition of viruses, bacteria, archaea, and fungi.

In Figure 1, we show a patient with widespread mpox-like lesions across the body during the ongoing mpox subclade Ib outbreak in the Eastern Democratic Republic of the Congo. Written consent to publish the picture was obtained. The picture was taken by a field researcher and had the face of the patient cropped.

### 3.2. Trans-Kingdom Taxonomic Annotation

**Figure 2:**
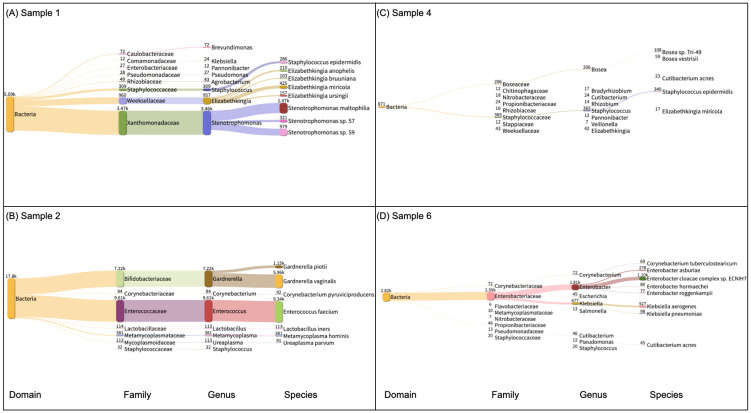
DNA-to-DNA bacterial taxa classification of mpox lesion samples. Trans-kingdom taxonomic composition of bacterial reads from sample 1 (**A**), sample 2 (**B**), sample 3 (**C**), and sample 2 (**D**) as per the Kraken2 classification. In the x-axis, we show the precise lineage of each bacterium.

**Figure 3:**
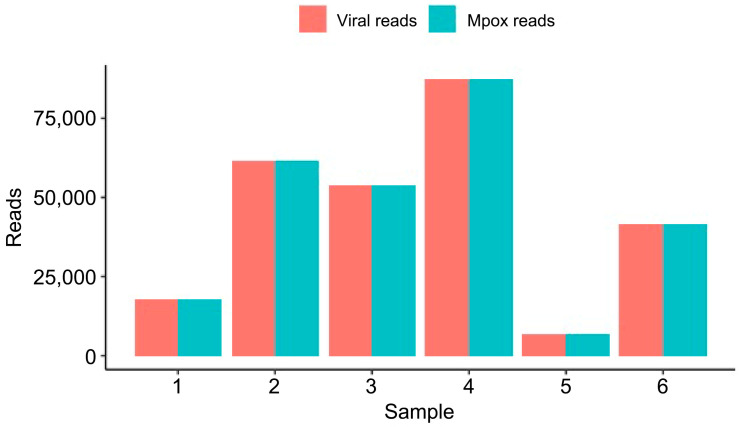
Viral taxonomic classification of mpox lesion samples.

First, we analyzed the bacterial DNA classified in each sample (Figure 2). Samples 3 and 5 had 74 and 13 reads classified as bacteria; thus, we opted to leave them out of Figure 2 due to low bacterial reads. On the other hand, Samples 1, 2, 4, and 6 had 5.009 K, 17.8 K, 874, and 2.82 K DNA reads classified as belonging to the bacterial domain. No bacterium was found in common across all four samples. *Staphylococcus epidermidis* was found in Samples 1 and 4 with 286 and 340 reads, respectively. Moreover, in Sample 1, a total of 3.46 K reads had their DNA classified as belonging to the Stenotrophomonas genus (*S. maltophilia, S.* sp. 57, and *S.* sp. 59). Sample 2 had 5.96 K reads of *Gardnerella vaginalis* and 9.34 K of *Enterococcus faecium*. Sample 6 had 1.81 K reads classified in the Enterobacter genus with 1.10 K reads belonging to the bacterium *Enterobacter cloacae complex* sp. ECNIH7. We also explored the DNA-to-DNA taxonomy classification of the six samples across fungi. In Sample 2, which had 194 reads classified as fungus, 184 reads were classified as belonging to the DNA of *Nakaseomyces glabratus*. The remaining fungal reads classified in the other samples were not mapped to any specific organism. Finally, we explored the DNA-to-DNA taxonomy classification of the six samples across viral species (Figure 3). The number of DNA reads classified as MPXV reads was above 99% of the total viral reads across the six samples.

We isolated the bacterial domain of the Kraken2 taxonomy classification of Sample 1 (Figure 2A), Sample 2 (Figure 2B), Sample 4 (Figure 2C), and Sample 6 (Figure 2D). The thickness of the horizontal links between taxonomical ranks represents the number of reads. The plots were generated with Pavian (version 1.0, running on R version 4.1.2). The taxonomical ranks displayed are domain, family, genus, and species. The numbers shown in superscript on the left side of each taxonomical rank represent the total number of reads whose DNA was classified.

The taxonomic distribution of viral DNA reads was classified using Kraken2 [28]. We show in the *x*-axis each sample and in the *y*-axis the absolute number of viral and mpox reads classified in a DNA-to-DNA approach.

## 4. Discussion

In our study, we determined the trans-kingdom spectrum of microorganisms found in pustule lesions of suspected mpox patients with metagenomic sequencing using DNA-to-DNA and DNA-to-protein taxonomical classification approaches. Each sample presented in this study provided reads classified as viruses, bacteria, and fungi, which all might play important roles in contributing to pathogenicity.

Beyond causing skin rash and lesions, mpox can trigger a strong effect on the host immune response, characterized by impaired Natural Killer cell function, lymphopenia, increased antibodies, increased blood monocytes and granulocytes, immune evasion, cytokine storm, inhibition of the host complement system, and antibody-dependent enhancement [29]. Therefore, during mpox infection, infected individuals may be more prone to bacterial, viral, and fungal infections as the immune response is undergoing several rapid alterations in response to the initial pathogenic challenge (mpox virus) [30]. We argue that initial infection with mpox may have resulted in an immunocompromised state, facilitating further co-infection with the bacteria, viruses, and fungi identified in our analysis.

Before the widespread emergence of next-generation sequencing (NGS) technologies, the identification and interaction of microbes relied largely on in vitro culturing. Only within the last decade have the cost and barriers associated with NGS and metagenomic profiling decreased, allowing greater integration into clinical diagnostic practice [31]. Rather than isolating and culturing a single pathogen, metagenomic profiling provides insights into the viral, bacterial, and fungal makeup of a patient sample. The specificity of metagenomic profiling reduces false positives and provides a more sensitive, unbiased delineation of the causative pathogenic agents responsible for a given infection. Clinical metagenomics may also be leveraged to provide a tailored approach for treating a patient-by-patient disease spectrum, where co-infections often complicate the initial diagnosis [32]. Previously, Xu et al. [27] performed an observational study for face samples of acne vulgaris disease and reported the increased abundance of *Malassezia restricta* and *Cutibacterium acnes* in the non-inflammatory group with the aid of metagenomics analysis. That study also reported that *Staphylococcus epidermidis* and *M. restricta* have similar proliferation trends with *C. acnes* during the transformation from non-inflammatory to inflammatory lesions based on the correlation analysis.

In terms of viral classification, MPXV-classified reads were predominant across the six samples analyzed in this study. No other virus was found in any other sample. A low number of reads were classified as fungi. On the other hand, bacteria were found across samples. Despite not identifying a common bacterium in all samples, we observed Samples 1 and 4 having reads classified as *S. epipermidis*. There is evidence of this organism having evolved to not infect humans [33], thus configuring a commensal relationship. A total of 3460 reads of Sample 1 were classified as pertaining to the Stenotrophomonas genus (with predominance of the bacterium *S. maltophilia*). Skin infections of *S. maltophilia* have been classified as very rare among non-immunocompromised patients [34], which might suggest the patient acquired the infection when their immune system was already stressed. In addition, our taxonomic classification confirmed the presence of *E. faecium* and *G. vaginalis* in Sample 2. Both microorganisms have been reported to cause infection in humans [35,36]. In Sample 6, we identified bacterial reads classified as belonging to the Enterobacter genus, with more reads classified as belonging to the species *E. cloacae*. Despite the lack of evidence of direct diseases caused by *E. clocae*, this pathogen is commonly found in nosocomial infections, with a high rate of mortality associated with the Enterobacter genus [37,38].

Limitations of our study include the possible sample bias and lack of sequence information for control skin samples as comparators for the mpox pustules. In the viral classification step of our study, only MPXV was identified, as it was the only virus detected among all classified viral reads. Our methodology relied on long-read DNA sequencing, which is specifically designed to sequence DNA molecules. Consequently, RNA viruses, whose detection requires RNA sequencing or prior reverse transcription of RNA into cDNA, were not captured in our analysis. Moreover, the general lack of health infrastructure in the South Kivu province limited testing/sampling capacity, and ongoing armed conflict in the region reduced our ability to test for HIV, potentially introducing confounders and influencing the immune status of study subjects. Finally, despite having identified opportunistic organisms, such as *S. maltophilia*, which generally causes infections in already immunocompromised individuals, the lack of longitudinal data in our study makes it difficult to speculate on the trans-kingdom spectrum of patients prior to their MPXV infection.

## 5. Conclusions

In conclusion, our trans-kingdom analysis identified potential bacteria with known and unknown pathological functions coexisting within mpox virus skin lesions. These samples were collected during an ongoing MPXV outbreak in the South Kivu province of the DRC. The findings from this study should serve as a warning sign for public health authorities that additional microorganisms may be contributing to the disease severity and transmissibility observed during the subclade Ib outbreak. Furthermore, our findings underscore the importance of continued genomic surveillance in mpox endemic regions and highlight the need for improved routine STI screening in these settings. Further investigation is needed to determine if human populations living in different geographical locations have similar or different flora in mpox lesions.

## Figures and Tables

**Table 1:** List of the six samples with virus names and accession numbers, along with the collection dates submitted to GISAID.

S. No	Virus Name	Accession Number	Collection Date
1	hMpxV/DRC/CRSN-1/2023	EPI_ISL_19004044	2 November 2023
2	hMpxV/DRC/CRSN-2/2023	EPI_ISL_19004045	15 November 2023
3	hMpxV/DRC/CRSN-3/2023	EPI_ISL_19004046	23 December 2023
4	hMpxV/DRC/CRSN-4/2023	EPI_ISL_19079342	23 December 2023
5	hMpxV/DRC/CRSN-5/2024	EPI_ISL_19079343	6 January 2024
6	hMpxV/DRC/CRSN-6/2024	EPI_ISL_19079344	14 January 2024

**Table 2:** Metagenomic sequencing and taxonomic annotation of six mpox lesion samples.

	Sequencing				Trans-Kingdom Composition		
Sample	Raw Reads (Million)	Total Bases (Gb)	Passed Bases (Gb) ^1^	Mean Read Length, stdev, IQR	Bacteria/Archaea (Reads)	Fungi (Reads)	Viruses (Reads)
1	0.964	3.18	2.43	3430.63, 1464.47, 1695-4112	5009	25	17,896
2	2.07	7.02	6.33	3910.66, 1593.1, 2316-3765	17,800	194	61,808
3	3	11.18	10.45	3159.89, 1413.37, 2825-4776	74	14	53,868
4	1.29	5.13	4.59	3741.09, 1302.62, 2863-4601	874	15	87,600
5	1.96	4.64	3.80	2715.25, 1119.55, 1922-3308	13	1	6821
6	1.58	5.23	4.63	3482.13, 1316.61, 2533, 4296	2820	11	41,620

^1^ Q score ≥ 8.

## Data Availability

The original contributions presented in this study are included in the article. Further inquiries can be directed to the corresponding author.
